# The victimisation of farms in Ireland: fear of crime, social isolation and crime prevention

**DOI:** 10.1057/s41300-022-00153-1

**Published:** 2022-06-18

**Authors:** Margueriete O’Brien, James Windle

**Affiliations:** Department of Sociology and Criminology, University College Cork, Askive, Donovan’s Road, Cork, Ireland

**Keywords:** Elegant security, Farm crime, Fear of crime, Ireland, Routine activity theory

## Abstract

This paper explores farmer’s experiences of crime and their attitudes towards crime prevention in one rural hinterland. Farmer’s attitudes about safety and crime present a dichotomy: fear of victimisation was relatively high, yet few participants reported having been victimised, and there was a perception that agricultural crime was high in Ireland but low in their locality. Feelings of insecurity were partly influenced by the closure of rural Garda stations and uneven distribution of information technology. Participants were most concerned with theft of small machinery, violent coercion connected to fraudulent work, illegal dumping and trespassing, rather than thefts of expensive machinery and livestock. Participants reported being unable to afford some crime prevention measures and/or having insufficient time to implement them. The paper concludes by highlighting the relevance of Farrell and Tilley’s (2020) concept of elegant security to farm crime and discussing the role of community policing.

## Introduction

The literature on agricultural crime in Ireland runs to three studies: a theoretical paper (Windle 2022), a qualitative case study (Bowden and Pytlarz 2020; Pytlarz and Bowden 2019) and a larger survey (Walsh and Walsh 2017). While criminological research is relatively new in Ireland (Lynch et al. 2020; Windle et al. 2022) this lack of academic attention remains surprising: there were approximately 137,500 farms operating in Ireland in 2016, with around 10% of the working population employed in the agri-food sector (Teagasc 2020). Furthermore, the media, including specialist farming publications, commonly report that theft is increasing and becoming more violent (see Bowden and Pytlarz 2020; Windle 2022).

Theft from, and damage to, farms not only present significant economic costs,^1^ which can be passed down to consumers, but fear of crime can impact quality of life. Research in the UK and USA show how farm crime can place farmers under stress, prevent families from taking vacations and motivate some to change career (Mears et al. 2007; NRCN 2018; Smith 2020). In Ireland, journalists and politicians have reported that fear of victimisation has reduced community cohesion (Hamilton, 2019) and some farmers have suffered depression after repeated incidence of burglary and theft (Danaher 2019; see Windle 2022).

As the literature on farm crime in Ireland is scarce, this article presents an exploratory case study of one rural hinterland, consisting of seven semi-structured interviews with farmers, designed to explore farmer’s perceptions and experiences of victimisation and crime prevention. While a much larger sample is needed to generalise the findings to the wider Irish population, the study represents a first step towards understanding offences committed against Irish farms and the security measures farmers take to prevent victimisation. The current study is designed to inform a larger study which will give a voice to the social reality of farming in Ireland.

## Review of farm victimisations studies

This literature review focuses on the level and scope of farm crime and reported crime prevention measures. As just one farm victimisation survey has been conducted in Ireland (Walsh and Walsh 2017), the review also draws from studies conducted in other countries. While these international studies offer important insights and benchmarks for the current study, Irish farms tend to differ from these jurisdictions in terms of size, management structure and terrain. As such, their relevance for Ireland is limited and further research in an Irish context is needed (Windle 2022).

### Extent of victimisation

Previous farm crime surveys, across a number of different countries, have found relatively high rates of past-year victimisation. These have ranged from: 30% in a Swedish study (Ceccato 2016); 50 and 62% in an American study (Mears et al. 2007); 50 (Morris et al. 2020), 62 (Smith and Byrne 2017) and 82.5% (Sugden 1999) in three British studies; and 87% in an Australian study (Barclay and Donnermeyer 2002). Another American study found that 33.4% of farms had been the victim of theft (McIntyre et al. 2017).

In the Australian and Swedish studies, the most frequently reported crimes were theft of fuel, livestock, tools and equipment (Barclay and Donnermeyer 2002, 2011; Ceccato 2016). The theft of small tools where the most common offences in an American study followed by chemical/fuel theft, breaking and entering, and large equipment theft. The offences that most concerned farmers were, however, machinery theft, illegal dumping, burglary of farm buildings and vandalism. The theft of large plant was reported as rare but carried significant economic consequences (Mears et al. 2007). In a recent British study, theft of farm machinery and livestock were the most reported crimes (Morris et al. 2020). A survey by the British National Rural Crime Network, however, found the most commonly occurring crimes against ‘specific rural businesses’ (mainly but not limited to farms) were fly-tipping, wildlife crime and theft of agricultural machinery/equipment (NRCN 2018).

In a farm victimisation survey conducted in Ireland, by Walsh and Walsh (2017:4), 75% of the 861 participants reported having been victimised, of which 41% had experienced more than one incident. The most common offences reported were: theft, vandalism, criminal damage and trespass, assault and fraud. The items most often stolen were: fuels and oil, followed by tools and machinery and equipment. Other items stolen, reported by less than 10% of participants, were: livestock, vehicles, building materials, crops and fodder and chemicals. Only 3% of thefts involved the threat or use of violence.

### Routine activity theory and farm crime

Farms can make attractive targets. They often consist of large areas of land situated in remote locations, shielded by natural barriers from would-be guardians. Many goods found within farms are attractive to potential offenders, ranging from portable tools which can be quickly sold to very profitable machinery (Donnermeyer and Barclay 2005).

Almost all studies which have explored why farms are victimised have been influenced by, or exhibit elements of, routine activity theory (Cohen and Felson 1979; see review in Donnermeyer 2018). For example, Barclay and Donnermeyer (2002) surveyed 393 farmers in Australia to investigate how the physical farm environment can influence victimisation. They found that: the further away a farm was from an urban area, and the hillier the terrain, the more likely it was to have experienced livestock theft; farms and farm buildings visible from houses were associated with lower-levels of breaking and entering, trespassing and theft; and farms surrounded by dense cover (i.e. trees, bushes) were more likely to report higher levels of stock theft and illegal trespass. These findings are summarised by Barclay and Donnermeyer (2002: 58) as ‘the ease of accessibility makes a farm a more suitable target, as does the lack of sufficient guardianship’ and, in a later paper: as ‘visibility decreased, crime increased’ (Barclay and Donnermeyer 2011: 14; also Ceccato 2016).

Other studies have found that being situated near urban areas and main roads increases the risk of victimisation (i.e. Bunei and Barasa 2017; Mears et al. 2007). While Smith and Byrne (2017) found no evidence linking victimisation to proximity of urban areas, they identified repeat victimisation as more likely in isolated farms. McIntyre and colleagues (2017) found that larger farms experienced higher rates of victimisation. Mears and colleagues (2007) found that target hardening efforts were dependent upon the physical environment, especially the presence or absence of capable guardians. Harkness (2017) found that having neighbours who monitored for unusual activity was a most effective crime prevention measure and that the presence of non-residential farm workers could increase opportunities for theft.

Consistent with the macro-level element of routine activity theory (Cohen and Felson 1979), some studies have shown how changes in farming and transport infrastructures have increased opportunities for crime (see Barclay and Donnermeyer 2011; Bunei and Barasa 2017; Sugden 1999). In Ireland, Gardaí (Irish police force) and politicians have argued that increases in agricultural theft followed improvements in the motorway infrastructure, which allowed urban gangs to travel into the countryside (see Carswell 2017; Houses of the Oireachtas 2019). A review of Gardaí reported crime data found some support for this proposition with an average 10% ‘rise in the burglary rate (or equivalently, five burglaries) in the same year a motorway is placed within 30 km’: although burglary rates plateaued after the first year (Agnew 2020).

### Crime prevention measures

A consistent finding across the international literature is that farmers tend to be complacent about security and that crime prevention is seldom at ‘the forefront of the minds of farmers’ (Jones 2010: 40; also Harkness 2017), often because farmers lack time and resources (Windle 2022). Several studies have found that many employ only basic security measures, often limited to locks and dogs (Harkness 2017; Harkness and Larkins 2020; Holmes and Jones, 2017; Smith and Byrne 2017; Jones 2010; in Ireland, Walsh and Walsh 2017). As Harkness (2016:104) notes in an Australian study:

Many opportunities are provided inadvertently for thefts to occur, such as: irregular livestock counts; tractors which are keyed alike; keys left in ignitions of unlocked vehicles and machines; sheds left unlocked; and machinery left near roadsides out of sight of the farm house.

### Reporting victimisation to the police

Several studies have found that farmers are slow to report crime to the police, although reporting varies by offence (see Donnermeyer and Barclay 2005; Mears et al. 2007). For example, an American study found that theft was reported to the police 58% of the time, but illegal dumping only 21% of the time (McIntyre et al. 2017) while almost all respondents to a British survey had reported farm business crime to the police (Morris et al. 2020). The Irish farm victimisation survey found that just 41% of theft cases were reported: the crimes most likely to be reported were machinery and vehicles thefts, and robbery (Walsh and Walsh 2017).

There is a great deal of consistency between studies, across a number of different countries (Windle 2022) including Ireland (Walsh and Walsh 2017), regarding why farmers choose to not report crimes. Farmers often report that: a level of victimisation is inevitable (Holmes and Jones 2017); some crimes are too difficult to prove and/or there is uncertainty that a crime occurred (i.e. strong winds could have broken fences) (Donnermeyer and Barclay 2005; Harkness 2017; Harkness and Larkins 2020; Mears et al. 2007; Walsh and Walsh 2017); some crimes take time to notice, leading farmers to believe that too much time has elapsed to secure conviction/return property (Donnermeyer and Barclay 2005; Harkness and Larkins 2020); police do not understand farming (Donnermeyer and Barclay, 2005; Walsh and Walsh 2017); victims want to avoid angering the community by informing a neighbour to the police or harming the suspects family (Donnermeyer and Barclay 2005; Walsh and Walsh 2017); victims fear retribution (Harkness 2017; Harkness and Larkins 2020; Walsh and Walsh 2017). In addition, the Irish Farmers Association claimed that many farmers stopped reporting crime to the Gardaí during the 2008–2012 economic recession because they felt abandoned by the closure of many rural Gardaí stations (Gallagher 2018b).

## Method

As farm crime is understudied in Ireland, the present study is designed as an exploratory foray to provide a foundation for a larger study. Farm crime is here defined as a subcategory of rural crime focused on the victimisation of farms (Anderson and McCall 2005) or crimes committed by farming enterprises (Ceccato 2016).

Semi-structured interviews were conducted with seven farmers living and working in one anonymised hinterland in County Cork. The town is situated around 60 km from Cork City, has a population of approximately 5,000 spread between a medium-sized town and hinterlands. Agriculture (including a large concentration of cattle farming) and tourism are the areas main industries, although there is a growing technology sector. All participants were aged 18 years or over, full- or part-time farmers and owned or rented a farm-holding. Three participants were aged in their 30s, three in their 50s and one in his 60s: two were female and the remaining five were male. All participants operated dairy farms. All but one of the farms were relatively small family-run businesses who employed contractors or staff on a seasonal basis: the smallest farm was 50 acres concentrated in one area. The one larger farm (350-acres across several sites) employed one full-time and three part-time seasonal staff.

The study employed convenience sampling. Participants were known to the first author. While convenience sampling was appropriate due to the challenges of recruitment and the exploratory nature of the study, it is acknowledged that this sampling method injects an element of bias (Boeri and Lamonica 2015). A larger study, using a more representative sample and/or cases from different areas, could uncover different findings.

Interviews were conducted between June and September 2021 and lasted on average 50 min. Participants were not paid. Ethical clearance was provided by our institutions Research Ethics Committee. Due to Covid-19, most interviews were conducted via Microsoft Teams, although two participants had reservations about using digital technology so requested telephone interviews. These were shorter and less detailed than the virtual face-to-face interviews. This is unsurprising considering the centrality of non-verbal exchanges and body language to generating interview data (see Thunberg and Arnell 2021).

### External-insider researchers

Farmers are notoriously reticent about talking to researchers: 'they are … somewhat taciturn and reserved, particularly to outsiders'. As such being known to the local community can be helpful (Sugden 1999: 29; also Bowden and Pytlarz 2020; McIntyre et al. 2017; Windle 2022). Some participants agreed that they would be slow to discuss their experiences of victimisation with strangers and the current study was possible because the researchers are not outsiders. Both married into established farming families and may best be described as ‘external-insiders’: while born outside of rural Ireland, our position in extended kinship and friendship networks supported access, recruitment and rapport building, provided legitimacy (i.e. we can talk confidently about farming and rural issues) and aided interpretation of the data. For example, we understood slang, jokes, ‘nonverbal gestures of embarrassment and discomfort’ or unusual or potential untrue responses (Chavez 2008: 479; also Merriam et al. 2001).

Insider researchers have, however, been ‘accused of being inherently biased, and too close to the culture to be curious enough to raise provocative questions’ (Merriam et al. 2001: 411). To limit bias and facilitate greater objectivity, the first author conducted and transcribed the interviews. The anonymised transcripts were then analysed separately by both authors and space was provided in supervision meetings to reflect upon any biases.

## Results

The objective of this study was to explore farmer’s experiences as victims of crime and their attitudes towards crime prevention. The farmer’s attitudes about safety, security and the threat of victimisation were complex and present a dichotomy: fear of victimisation was relatively high, yet few participants reported having been victimised, and there was a perception that agricultural crime was high in Ireland but low in their locality. This finding parallels a case study in another part of Ireland by Pytlarz and Bowden (2019). Participants were asked questions specifically about farm crime; however, many drifted into discussions around wider rural crime issues. This may highlight the difficulties of detangling farm crime from the wider category of rural crime.

Source: CSO (2021).

**Fig. 1:**
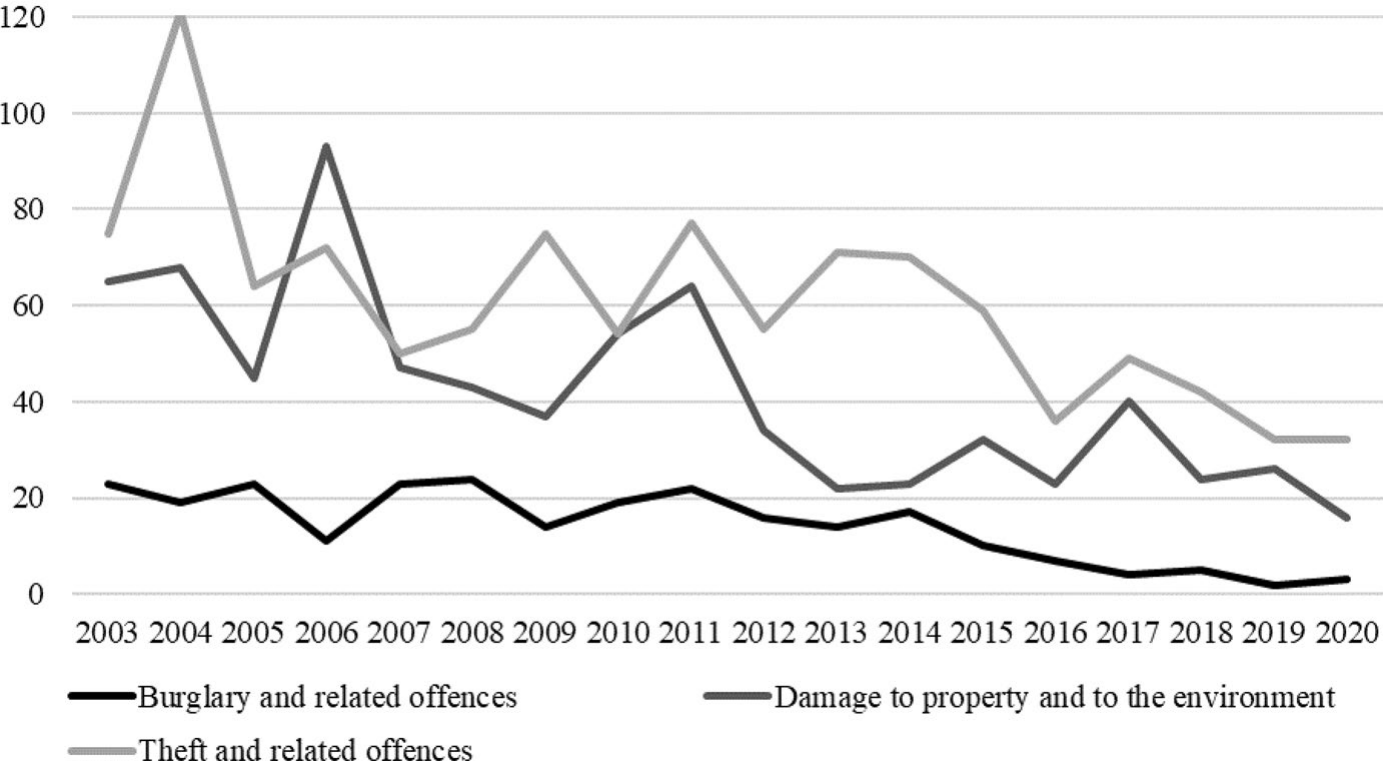
Local Garda station reported crime rate

Overall, there is a disparity in participant’s responses: they reported living in a low crime area *and* being fearful of crime. Officially, the local crime rate is relatively low. Between 2003 and 2020, the average number of burglary and related offences reported to the towns Garda station, per 100,000 of the national population, was 0.28 compared to 2.39 for one Garda station in the nearest inner city area (CSO 2021; see also Houses of the Oireachtas 2019). Furthermore, the number of theft, burglary and property damage offences appear to be declining (Fig. 1). This data must, however, be taken with caution: it only represents offences reported to the Gardaí, farm crimes are not counted as distinct categories and, at the time of writing, the data were under review for inconsistencies in reporting practices (CSO 2021).

Official data aside, all participants felt that rural crime had risen during this period, although most seemed to talk abstractly about national increases while acknowledging that crime was low in their vicinity:

It has never been as big as it has been in the last fifteen years, one hears stores about years ago, where there was no such thing as rural crime, people used to come into the yard to give a hand with something rather than to case the place (R.2)

R.6 felt that crime was low in rural Ireland until the start of the Great Recession (2008–2012), when, he felt, economic insecurity and joblessness drove many towards crime. R.1 felt that farm crime increased from the early 2000s because there was more to steal and reduced guardianship; an observation aligning with routine activity theory (Cohen and Felson 1979):

Around the farmyard [pre-2000s], there was very little to rob, there was no quads, probably only one tractor. Whereas now, old fashioned labour is gone, most farmers have everything, from big machinery and, quad bikes to valuable cattle. With no Gardaí around thieves become more confident and daring. Now, everybody has everything, like diesel tanks, or house heating oil (R.1).

### Victimisation

Most participants declared that they had not been the victim of farm crime, although some were unsure what could be defined as farm crime: R.1 felt she had not been a victim of farm crime even though her farmhouse had been burgled; some felt that smaller thefts should not be counted; and R.2 called illegal dumping on farmland a ‘pretty minor incident’ which he would ‘feel a bit foolish’ calling farm crime.

Participants appeared most concerned with thefts of small machinery, fraud, illegal dumping and trespassing, rather than thefts of expensive machinery and livestock which they felt was rare. Violent coercion connected to fraudulent work, often directed at older farmers, was a particular concern of many participants (also Pytlarz and Bowden 2019):

With older farmers it is money, fellas coming in to do a job, charging exorbitant prices for the job and threatening the farmers if they won’t pay up (R.2).

R.6, an elderly farmer, reported how aggressive door-to-door selling and theft were related:

The lads that called here trying to sell me stuff were very aggressive, and I didn’t buy from them. Anyway, that night some stuff was taken from the yard (R.6).

R.2 found illegal dumping ‘very frustrating’:

People dropping rubbish, armchairs, black bags, fridges …. if the cattle got at it, well, it could kill them. I’m not too far from the town you see… they know as a busy farmer I can’t man my property all the time, it’s easy to guess when I am around and when I’m not (R.2).

Many participants believed that crime rates were higher in other parts of rural Ireland. Some felt that the isolation of their farms prevented more expensive items being stolen: all owned large machinery and livestock, which they believed were unsuitable targets for theft due to geographic location and because such theft needed specialist knowledge:

You hear of small things, see they can’t take big things, you see I think they could hardly drive them. I don’t think the animals need safeguarding, we are down beside the water here, so we are o.k. (P.5). Look the newspapers are always on about Ireland’s good roads, well not around here. I suppose that is a good thing, it might slow criminals down (P.6).

While the belief of a ‘rural idyll’ (Jones 2012), protected from crime by nature and infrastructure, holds a certain romantic sway, an expectation that crime occurs elsewhere can cultivate ambivalence towards crime prevention measures (Harkness 2017; Holmes and Jones 2017). For example, R.2 reported that:

I wouldn’t take out the tractor keys because it is old, if it was newer I probably would, but my tractor is not worth stealing. They would be better off robbing something of more value (R.2).

This perception of tractor value and attractiveness may be correct. It may also, however, rest on a faulty assumption about the choices potential thieves make and, their perceptions of risk and reward. Furthermore, as some participants noted, thieves are not always looking for tractors but tractor parts. That is, they may not want to drive machinery away but strip valuable parts (see Windle 2022).

### Vulnerability

Respondents generally viewed farming as a lonely life and talking about crime occurring elsewhere unleashed a sense of vulnerability and unease:

For me it’s so far so good… It’s a national concern – we hear about it all the time**…** it would make you think about it all the same (R.5).

Participants identified two characteristics which make some farmers more vulnerable to crime: living alone in isolated areas and being older. The older farmers tended to be more openly concerned about potential victimisation than younger participants and appeared more aware of their vulnerability. For example, one farmer in his 30s reported being unconcerned about his safety but felt that older farmers were ‘prime targets’:

I am young so no nothing phases me. Maybe if I was in my 40s with kids I might think differently (R.2).

R.5, also in his 30s, reported feeling safe, but worried for an elderly relative who lived alone. R.6 made an explicit link between being an older farmer and potential victimisation.

I’m a sitting duck, living out here rurally makes one feel very unprotected. Whether I’m a farmer or a fisherman doesn’t matter…. when lads come round here looking for trouble, they see a quiet road, a house in darkness and no-one around, they’re in … I am a lot more cautious now as an older man living alone, I don’t even think about doing things the same as before … I do live in fear now most of the time. I don’t say that to my daughters of course, or they would think I cannot take care of myself but I am frightened alone here at night (R.6).

This quote shows how the fear of crime can be constricting and constraining (see Smith 2020; Yarwood and Gardner 2000) and prevent farmers from doing things which they value, further isolating already isolated individuals.

There are an estimated 41,200 farmers aged over 65 in Ireland (Department of Agriculture, Food and Marine 2021). The Irish Farmers Association (2019) have suggested that older farmers are more vulnerable to crime, and there is some support for this proposition in the research literature. For example, Mawby (2015) found evidence that ‘distraction burglars’ predominantly target older populations, although this area has not been researched in an Irish context. Irish research has, however, found that older people are more fearful of crime, regardless of their actual risk of victimisation (Northern Ireland Perceptions of Crime Survey, 2016/2017).

### Social exclusion and fear of crime

Two issues emerged during interviews as affecting feelings of safety: the closure of rural Garda stations and the uneven distribution of information technology. Both centred on the unequal streamlining of public services and meshed into a general feeling of disconnect from the state and perception that their rural community is forgotten by, and excluded from, the predominately urban government. Such perceptions have been reported elsewhere in Ireland (Houses of the Oireachtas 2019; Pytlarz and Bowden 2019) and Australia (Anderson and McCall 2005).

None of the participants professed faith in the Gardaí to prevent or investigate agricultural crime. Several were angry at the closure of rural Garda stations^2^:

The closing of rural Garda stations is a disaster, it has left parts of this country in a pure state, a place where thugs can come and go as they please, nobody is safe without the Garda stations (R.2). Most of the smaller Garda stations have closed, you can ring the bigger stations, but they have no interest in what goes on out here, they are after the bigger fish in the towns (P.6).

Others felt that the Gardaí did not care about rural Ireland:

The Gardaí are not interested in what happens around here. That is local knowledge. Look around you, we are on our own here. They want to stay around the towns and be the big boys (R.4, also R.2). I made it known to the Gardaí that there were fresh tyre marks in a field where I keep beef cattle and the gate was tampered with. A Garda did call after three days, they didn’t care that my land was violated, they didn’t worry about me, I mean it’s up to us down here to protect ourselves, while the Gardaí protect the towns, and at our own risk (R.5).

Some participants were slow to report smaller crimes to the Gardaí for four reasons; which parallel findings from studies conducted in Australia, the UK and USA, and Ireland, as discussed in the literature review section of this article. First, four participants (R.2, R.5, R.6, R.7) felt that reporting was pointless because the Gardaí lacked interest in agricultural crime: R.2 had failed to report illegal dumping because ‘what Garda is going to bother coming out to investigate something as minor as a few black bags of rubbish dumped on my land’, while P.6 felt that the Gardaí would ‘only laugh at me for wasting their time’ for reporting theft of tools. Second, three participants (R.2, R.4, R.5) identified the innate secrecy of rural communities: R.2 felt that ‘farmers often keep things to themselves as they are afraid of what the neighbours might say’, while R.5 reported how:

Older farmers … won’t talk about things and if they get robbed, they are too embarrassed to say because they know they should have things locked down, but that’s the way of the older generation --- guilt (R.5).

Third, R.5 felt that agricultural thefts were often too difficult to prove:

I never hear about anyone being up in Court for crime on farms, what I do hear from other lads is that the Gardaí usually say that it’s too hard to prove (R.5)

Fourth, R.2 felt that farmers often avoided reporting due to repercussions: ‘people are afraid to report in case whoever robbed them comes back and beats the shit out of them’.

P.1 and P.3, however, reported a more positive experience with the Gardaí after their farmhouse was burgled and felt the Gardaí were less available than in cities due to insufficient resources rather than lack of interest from individual Gardaí (see also Mawby 2009; Yarwood and Gardner 2000). While Harkness (2017), in Australia, and Mawby (2009), in the UK, found that farmers who had been a victim of theft had fewer positive beliefs about the police, participants in the present study with previous encounters considered the Gardaí response to be more proactive. Farmers with little experience of victimisation were the more critical of the Gardaí. That is, farmers who had reported a crime to the Gardaí were more satisfied with rural policing than those who had not reported a crime. This may suggest that it is less the actions of the Gardaí in responding, and more the lack of interaction between rural peoples and the Gardaí, which influences low levels of accountability and legitimacy in rural areas. This may point to the importance of community policing in reducing fear of crime.

### Crime prevention

Consistent with much of the rural crime prevention literature (Donnermeyer 2018), many responses aligned with routine activity theory. All participants identified the importance of what Cohen and Felson (1979) referred to as capable guardians. All believed that a visible presence on the farm could reduce victimisation; some had installed CCTV or sensor lights to facilitate guardianship; and several mentioned the importance of dogs as guardians, although some feared the dogs would be stolen.

Another form of guardianship mentioned was the local Text Alert system. This system was established by the Gardaí as a virtual neighbourhood watch whereby people could text the group about suspicious activities. Despite signs promoting the scheme placed around the area, none of the respondents knew how (or if) it worked. As such, there was minimal uptake of the scheme. It is possible that if residents were more concerned about local crime then they may have learnt how to use the Text Alert: that they had not engaged with the scheme may be indicative of the lack of felt fear.

Some participants’ identified social media platforms (i.e. Twitter, Facebook) as important crime prevention tools:

There is a dodgy looking vehicle driving around, it’s put up on social media straight away, and then it’s up to each man for himself to look after his stuff. We are like our own police group around here. That is down to the internet though. That must get better, if the government wants to invest in farmers, I say invest in rural broadband first (R.5).

Two points emerge from this quote. First, the rural/urban digital divide leaves farmers feeling even more isolated and, consequently, vulnerable to victimisation, a situation heightened by the perceived lack of police presence. Second, most participants identified crime prevention as something they alone were responsible for. Preventive tools, such as Text Alert, fed into the felt responsibilisation of rural communities and heightened feelings of isolation from the state (also Pytlarz and Bowden 2019; Holmes and Jones 2017):

It’s not just up to the Gardaí to keep us safe, we must be willing to guard ourselves and others... A lot of people are careless – leaving quads at the entrance to the farms is careless. We are the guardians over our own property. It is up to us to have things in place (R.1).

R.4 felt that farmers are complacent until they have been robbed and:

If fellas aren’t happy in their job, they won’t put in the effort to safeguard things. Some farmers never wanted to farm in the first place so don’t bother to put in the full effort (R.4).

Participants tended to understand the methods potential offenders could use and the measures they could employ to prevent victimisation. Implementing these measures was often, however, more difficult due to a combination of practical issues and scepticism about the effectiveness of crime prevention. Three participants believed that a certain amount of crime was inevitable:

Look if someone wants in, they will get in regardless of my efforts. The trick is I think, not to keep any money in the house, at least they won’t get at that if they come. I suppose I should have lights on and gates locked and dogs barking but I don’t (P.6).

But if thieves are going to go in and there is a diesel tank or a shed, they are going to get in anyway (R.3).

A certain amount of crime is inevitable it’s the nature of farming business, it’s too easy to access a farm, we can’t be watching things all of the time look (R.1)

Some reported making rational decisions to not prioritise preventive measures (see also Holmes and Jones 2017). Some were too busy:

I really know that I do not do enough but look who has time to do everything they should (R.5).

Its hard, I could be rushing in the evening, things might break down on the farm, a sheep might get out, then security becomes a thing at the back of the mind (P.7).

This is a farm, a working place so it’s hard to keep the routine of locking doors and gates, you know the farm is a busy place and we just wouldn’t find the time in the working day to lock the gate every time we drive through it, it’s just not practical (R.3).

Others identified the prohibitive cost of preventive measures. Some felt that only large-scale operators could afford hi-tech equipment and P.3 reported having ‘loads of lights everywhere’ to deter theft but leaving them on ‘seems a bit wasteful’.

Some farmers drifted into discussions of insurance when asked about crime prevention:

No matter how hard you try to secure the farm, they are still going to get you if they really want to, they know what you have that they can turn into quick money, so locks, bolts, gates or dogs won’t stop them, insurance is key (R.5).

This again suggests a practical inevitability about crime—that once you had done all you can, insurance provides a cushion against risk (see also Holmes and Jones 2017).

### Trust in the community

Again aligning with routine activity theory, the most agreed upon characteristic by respondents regarding safety was the importance of knowing neighbours:

A neighbour who was worried about his property recently rang four or five local farmers and we all kept an eye out (R.3).

The neighbours are close by. All we have to do is just look out the window there and you can see plenty houses. There is safety in that (R.1).

It is so important, it keeps me in contact with the neighbours, and keep check on what’s happening in the area it’s a safety net (R.5).

Several studies in Australia and the UK (i.e. Anderson and McCall 2005; Harkness 2017; Holmes and Jones 2017) have found lower levels of crime in farming communities with strong social ties linking neighbours together. The farmers in this study reported supporting each other practically by keeping an eye on each other's farms—acting as each other’s capable guardians—while avoiding discussing actual victimisation, so as to avoid ‘guilt’ (R.5):

I think farmers often keep things to themselves as they are afraid of what the neighbours might say (R.2; also R.7).

R.6 felt that some did not want to appear weak:

I suppose every man wants to be a big man and not come across as weak or frightened. A couple of the men I know for years do mention it from time to time, but not in front of the women, you know how skittish they are, no insult to yourself intended (R.6).

Studies in the UK have identified an ingrained culture of silence within farming communities (Smith 2010). As such, crime on farms can be hidden from view: a consistent theme in the international rural crime literature (Donnermeyer and Barclay 2005; Jones 2010; Holmes and Jones 2017). This silence may limit communal capacity for reducing repeat victimisation.

All but one participant reported an ‘alien conspiracy’ which identified much, if not most, crime as originating with ‘outsiders’ (see also Ceccato 2016; Holmes and Jones 2017; Morris et al. 2020). These outsiders were a constant source of anxiety. Travellers and immigrants were the ‘folk devils’ (Cohen 1972/2011) highlighted by some participants:

It’s always local people I employ, no, no, no strangers. All ourselves. I trust people that I know, but for those I don’t know I am half cautious coz you don’t know what they are looking for. If someone was calling you would be locking up stuff (R.5).

Whether the unease around these ‘folk devils’ is down to actual experience or bias is unclear, but some studies have shown how blaming and stereotyping ‘outsiders’ can ‘reinforce group solidarity’ (Donnermeyer et al. 2013:18) and it can be easier to identify threats as emanating from the abstract ‘other’ than known neighbours. Furthermore, some research outside of Ireland has cast doubt on the widely held belief that crime is carried out by outsiders with no attachment to the local area (see Jones 2010; Holmes and Jones 2017). Indeed, Smith and McElwee (2013:115) suggested that many ‘crimes, such as livestock rustling or theft of farm machinery often require the offender to possess insider knowledge and/or rural social capital’.

## Discussion and conclusion

A number of studies have suggested that opportunity theories may be particularly appropriate for the prevention of agricultural crime (Barclay and Donnermeyer 2002, 2011; Mears et al. 2007; Windle 2016, 2022), with a particular focus on the use of routine activity theory (Bunei and Barasa 2017; Donnermeyer 2018). Mears and colleagues (2007:136), for example, recommended a list of measures farmers can take to target harden and improve guardianship, including: locking chemicals and equipment in secure buildings; hiding/disabling equipment; locking gates; marking equipment and livestock; avoiding overstocking chemicals; and employing security personal. Clack (2020) and, Harkness and Larkins (2020) later added newer higher-tech options, such as ultraviolet etching, microdots or forensic water.

While these may all be useful, participants in the current study felt that even basic crime prevention measures can be too expensive and/or time consuming. A farmer struggling to justify the electricity costs of external lighting is unlikely to employ security personal: one participant laughed when asked if he employed farm labour.

Irish farms tend to be relatively small holdings and most Irish farmers are busy, often juggling on- and off-farm employment. As such, they must make rudimentary cost–benefit analysis when choosing whether to implement basic security measures. For example, it can be impractical to carry tools home every night or monitor farmland when fields are separated by 7 km, as was R.3 s farmland. For some, the time and financial costs of employing such measures can outweigh the potential cost of theft (Windle 2022), especially when living in communities where theft of expensive items is rare.

Even when farmers can target harden, few thieves will be dissuaded by basic padlocks. When Barclay and Donnermeyer (2002) cross-tabulated reported victimisation with 22 common security measures the only statistically significant measure found to reduce victimisation was a dog: something many participants in this study most feared being stolen. Barclay and colleagues (2002) acknowledged, however, that security measure effectiveness can be influenced by the motivation farmers have to use them.

Furthermore, the offence which most concern farmers was coercive or aggressive fraud and sales: when people conduct work for an (often elderly) farmer and demand more money than was agreed (or where no money was agreed), or when door-to-door sellers pressurise farmers to buy goods which may be stolen or faulty. This is a more difficult offence to prevent and is the more frightening because it is conducted at the farmhouse door.

Participant’s observations about the importance of neighbours for farm crime prevention align with previous studies conducted in Australia (i.e. Barclay and Donnermeyer 2011; Harkness 2017) and the capable guardian element of routine activity theory (Cohen and Felson 1979). In theory, Text Alert schemes provide a formal platform for extending guardianship and energising collective efficacy (Sampson et al. 1997). Several farmers in this sample did not, however, know how to use the scheme. Furthermore, if farmers feel guilt and shame at being caught in scams, they are less likely to tell others about their experience, thus weakening the influence of Text Alert schemes.

Farrell and Tilley (2020) recently proposed the need for ‘elegant’ security. That, as security measure affect quality of life, they should:

Preferably be ethical and unobtrusive, aesthetically neutral or pleasing, and the easy-to-use or default option … inelegant security can fall into disuse even if it prevents crime.

The Irish government and manufacturers of agricultural goods could make prevention easier for farmers who are often too overworked, and under too much financial stress, to undertake more than basic prevention themselves. For example, tractors and mobile agricultural machinery are seldom fitted with immobilisers as standard, let alone alarms (Windle 2022), yet research has shown that cars fitted with central locking, electronic immobilizer and alarms are ‘up to 25 times less likely to be stolen than those without security’ (Farrell et al. 2011: 21). As such, fitting appropriate security devices as standard would reduce machinery theft. Text Alert schemes may also need to be streamlined, made more efficient and as unobtrusive as possible to prevent inflating fear of crime. How to achieve more eloquent security on farms requires further research on what could work in an Irish setting.

The farmers in this case study felt isolated from the urban mainstream. Fear of crime was connected to feeling excluded and marginalised by insufficient Gardaí presence and responsiveness, augmented by insufficient broadband. The importance of police legitimacy to crime prevention is well documented (see Sweeney 2021) and the need for improved community policing in rural areas was a key recommendation of a Houses of the Oireachtas (2019: 37) committee on rural crime:

Community policing has been undervalued and marginalised in Ireland … Local communities take a significant degree of reassurance from seeing their local Gardaí. It helps to build relationships of trust, and assists in both the prevention of crime and in reducing the fear of crime, particularly as experienced by those in remote areas.

Participants who had dealt with the Gardaí had greater satisfaction with them than those who had not. Increasing the number of Community Gardaí in rural areas could rebuild the trust between state and rural communities. Having a Garda who can advise on basic crime prevention, and is known and trusted by the community, may help reduce fear of crime (see Sweeney 2021).

Finally, the lack of broadband in this rural area augmented feelings of exclusion and marginalisation from the urban mainstream and increased feelings of isolation, especially as some identified a role for social media in facilitating guardianship. The lack of broadband is, in some respects, a mirror to all other findings: fear seems to come less from actual experiences of crime and more from feeling excluded, and if preventive tools are available and cheap farmers are more likely to use them: chatting over social media may be as useful as Text Alert Schemes and more ‘eloquent’.

## Footnotes

1 An estimated €1.4 million worth of goods was reported to the Gardaí as stolen from farms in 2017 (Gallagher 2018a, b).

2 Over 100 Garda stations were closed for austerity measures during the Great Recession and many police resources were redeployed from rural to urban areas (Houses of the Oireachtas 2019).
